# A dominant set-informed interpretable fuzzy system for automated diagnosis of dementia

**DOI:** 10.3389/fnins.2022.867664

**Published:** 2022-08-01

**Authors:** Tianhua Chen, Pan Su, Yinghua Shen, Lu Chen, Mufti Mahmud, Yitian Zhao, Grigoris Antoniou

**Affiliations:** ^1^Department of Computer Science, School of Computing and Engineering, University of Huddersfield, Huddersfield, United Kingdom; ^2^School of Control and Computer Engineering, North China Electric Power University, Beijing, China; ^3^School of Economics and Business Administration, Chongqing University, Chongqing, China; ^4^Institute of Big Data Science and Industry, Shanxi University, Taiyuan, China; ^5^Department of Computer Science, Nottingham Trent University, Nottingham, United Kingdom; ^6^Cixi Institute of Biomedical Engineering, Ningbo Institute of Materials Technology and Engineering, Chinese Academy of Sciences, Ningbo, China

**Keywords:** clinical decision support, medical diagnostic systems, dementia, Alzheimer's disease, fuzzy systems, explainable AI

## Abstract

Dementia is an incurable neurodegenerative disease primarily affecting the older population, for which the World Health Organisation has set to promoting early diagnosis and timely management as one of the primary goals for dementia care. While a range of popular machine learning algorithms and their variants have been applied for dementia diagnosis, fuzzy systems, which have been known effective in dealing with uncertainty and offer to explicitly reason how a diagnosis can be inferred, sporadically appear in recent literature. Given the advantages of a fuzzy rule-based model, which could potentially result in a clinical decision support system that offers understandable rules and a transparent inference process to support dementia diagnosis, this paper proposes a novel fuzzy inference system by adapting the concept of dominant sets that arise from the study of graph theory. A peeling-off strategy is used to iteratively extract from the constructed edge-weighted graph a collection of dominant sets. Each dominant set is further converted into a parameterized fuzzy rule, which is finally optimized in a supervised adaptive network-based fuzzy inference framework. An illustrative example is provided that demonstrates the interpretable rules and the transparent reasoning process of reaching a decision. Further systematic experiments conducted on data from the Open Access Series of Imaging Studies (OASIS) repository, also validate its superior performance over alternative methods.

## 1. Introduction

Dementia is a syndrome in which there is deterioration in memory, thinking, behavior, and the ability to perform day-to-day activities (World Health Organisation, 2019). Among other types of dementia, Alzheimer's disease is the most common form of dementia and may contribute to 60–70% of cases. Despite the fact that young people could develop the condition, dementia mainly affects older people, which is not a normal part of aging. Dementia is one of the major causes of disability and dependency among older people worldwide. It can be overwhelming, not only for the people who have it but also for their carers and families. With around 50 million people having dementia worldwide, the total number of people with dementia is projected to reach 82 million in 2030 and 152 million in 2050 (Alzheimer's Research UK, 2018).

Although numerous new treatments are being investigated in various stages of clinical trials, there is no treatment currently available to cure dementia or alter its progressive course. Despite all of this, much can be offered to support and improve the lives of people with dementia and their carers and families. In practice, the diagnosis of dementia tends to take place late, possibly as a result of the lengthy manual diagnosis, being lack of training for frontline practitioners to make correct judgments, and/or the limited amount of primary care interactions (Cahill et al., 2008; Bradford et al., 2009) (with waiting lists up to 1 year weeks in the UK National Health Service NHS, 2019). Therefore, these strongly call for the early diagnosis in order to promote early and optimal management to preserve a high quality of life for as long as possible, which is also set by the World Health Organisation as one of the principle goals for dementia care (World Health Organisation, 2019).

Recent advances in machine learning have witnessed many successes in various domains including the healthcare industry (Chen et al., 2019a, 2022; Tachmazidis et al., 2020). The increasing availability of medical data in various forms such as imaging and electronic health records has facilitated the use of machine learning and data analytics to extract useful knowledge and patterns that support clinical decision making and enhance the effectiveness of healthcare delivery (Chen et al., 2020). While recent literature on machine learning for dementia research covers a wide range of novel ideas and points of view, classical ML approaches such as Support Vector Machine, Principle Component Analysis, and Random Forest remain highly popular while modern deep learning methodologies are also slowly being proposed with promising but mixed results (Tsang et al., 2019).

Among alternative techniques in machine learning, fuzzy systems represent knowledge explicitly *via* if-then production rules, supported by an inference framework that permits tracking back how an overall decision is finally made, thus enhancing the transparency and communication between end-users and the model. In addition, fuzzy models, which are constructed on the basis of fuzzy sets that allow gradual assessment of set elements, are able to deal with vague concepts that commonly exist in natural languages and clinical decision making. The reasoning of inference and the tolerance of imprecision have prompted its wide application in numerous medical applications (Mansoori et al., 2008; Kaiser et al., 2016; Chen et al., 2019b; Su et al., 2020a). However, the application of interpretable fuzzy systems in supporting diagnosing dementia barely appears in recent literature.

Inspired by the above observations, in working toward providing assistance for clinicians in practice, which typically requires support systems equipped with understandable rules and transparent reasoning processes, this article proposes a novel interpretable fuzzy inference system for the effective diagnosis of Alzheimer's Disease. It adapts the concept of dominant sets (Pavan and Pelillo, 2006) that arises from the study of graph theory and first identifies major patterns of patient data through dominant sets that may be regarded as a pairwise clustering problem. A peeling-off strategy is then used, which iteratively extracts from the constructed edge-weighted graph a dominant set, leading to the automatic generation of a fixed set of clusters, which are typically manually specified in conventional clustering approaches. Each dominant set is then converted into a parameterized fuzzy rule, which is further optimized in a supervised adaptive network-based fuzzy inference framework. The subjects researched in this study come from the renowned Open Access Series of Imaging Studies (OASIS) repository.

The remainder of this article is organized as follows. Section 2 first reviews related studies and then conducts an initial investigation of the data used. Section 3 proposes the dominant set-inspired fuzzy system. Section 4 analyzes the experimental outcomes. Section 5 closes the article with the conclusion and future study.

## 2. Background

### 2.1. Related literature

The increasing popularity of machine learning and its successes has gradually transformed medical research, clinical practice, and healthcare delivery. Particularly, research in dementia diagnosis typically involves a single or mixed use of a diverse set of features ranging from patient data modality such as demographic information and family history, neurocognitive measures designed to assess cognitive functions such as memory, learning, and language, to neuroimaging and biomarkers such as magnetic resonance imaging (MRI)/positron emission tomography (PET) scans and the cerebrospinal fluid (CSF) to measure protein levels. Binary predictions are often considered between being demented or not while many works also study the levels of severity (Fouladvand et al., 2019; Ieracitano et al., 2019; Jain et al., 2019; Ruiz et al., 2020).

On a broad level, the data used for classification are split between direct neuroimaging including MRI and PET scans, and tabular data. Direct interpretation of brain scan images has been shown to be effective in making classifications (Huang et al., 2019; Jain et al., 2019; Khan et al., 2019; Knox et al., 2021), particularly with the availability of imaging data from various (semi)-public sources including the OASIS and Alzheimer's Disease Neuroimaging Initiative (ADNI). Convolutional Neural Networks (CNN) are commonly used for feature extraction of images in multiple domains, including medical (Li et al., 2014) making them of interest in this domain. Recent CNN use has also shown potential for using Electroencephalography (EEG) recordings, converted into epoch sampled power spectral density images for diagnosis and produces promising results on per-epoch classification, but falls short when classifying individual patients on all epochs (Ieracitano et al., 2019). The addition of transfer learning to the toolkit increases options, particularly in areas where data is not available in large quantities for domains of interest, but are in related ones, where the convolution layers are transferred, replacing the fully-connected layers and training them using MRI/PET of AD related images (Jain et al., 2019; Khan et al., 2019).

The direct use of images can yet be computationally expensive and complex (Schlemper, 2019), which also makes the use of tabular data appealing and immediately usable against various techniques. Instead of working directly with raw images, this prompted some recent studies in the use of derived scan values, such as the volumetric data of hippocampus, ventricles, entorhinal, and fusiform gyrus from MRI measures, which may also be directly interpreted by physicians to explain the diagnosis in a clinical decision support system as an additional advantage. This is supported by popular imaging-oriented repositories such as OASIS and ADNI, which also provide readily available tabular data from various data modalities including imaging. In recent literature, Bucholc et al. (2019) considered the mixed use of 66 features from ADNI as potential predictors of cognitive decline associated with AD including clinical measures, risk factors as well as derived neuroimaging measurements from MRI and PET scans, with the performances varying depending on the combination of features used, resulting in the best accuracy of 83% using SVM. A similar best performance is also achieved in Stirling et al. (2020) while applying a self-organizing fuzzy classifier. The promising results achieved have also led to a prototype design of a clinical decision support system that incorporates the computational approach for tabular data, which is ready to be implemented in clinical practice (Bucholc et al., 2019). The OASIS MRI data that has typically been studied by directly working with raw imaging data has also been investigated on its associated tabular data out of derived imaging features in Bansal et al. (2018), with the J48 decision tree achieving the best result compared with Naive Bayes, Random Forest, and Multilayer Perceptron.

From techniques perspective, while recent literature covers a wide range of novel methods and points of view, a recent survey by Pellegrini et al. (2018) reviews over hundred relevant studies, with most assessing Alzheimer's disease vs. healthy controls using support vector machines (SVM), among other popular techniques such as random forest and neural networks. A comparative study is also conducted by Miah et al. (2020), which compares several popular machine learning techniques in identifying dementia from clinical datasets, with SVM and random forest typically achieving the best results. These are in line with the findings summarized in one of the latest surveys (Tanveer et al., 2020), which has concluded that the SVM-based models have been widely used for Alzheimer's disease showing its robustness, as techniques like artificial neural networks suffer from the drawbacks of local minima. The abundant usage of SVM also stems from the fact that it is easier to interpret in comparison to deep neural networks, which may give promising results by modeling highly complex data, but act as black box models (Mahmud et al., 2018), thus also calling for research in the clinical interpretability of machine learning models.

Through natural language for expressing terms to conjugate mathematical formalism and logical inference with human-centered interpretability, fuzzy rule-based systems have been universally acknowledged as valuable tools to model complex phenomena while preserving a readable form of knowledge representation (Alonso et al., 2015), making them specifically suitable in real-world applications where human beings are in charge of crucial decisions (Tahmasebi and Hezarkhani, 2012; Chen et al., 2016; Su et al., 2020b; Consiglio et al., 2021). On the basis of fuzzy sets and fuzzy logic that support the working with vague concepts typically existing in linguistic communications as well as the imprecision and uncertainty embedded in the collection of medical data (e.g., inaccurate test results), fuzzy systems have been developed and applied in numerous healthcare sub-areas that support clinical decision making through learned fuzzy medical knowledge.

For instance, a Takagi-Sugeno-Kang (TSK) fuzzy system (Jiang et al., 2020) that combines multiple-source transfer learning and manifold regularization learning mechanisms is effective in identifying EEG signals while achieving good interpretability that can be comprehended by medical experts. A Naive Bayes approximation based fuzzy system by Pota et al. (2017) was proposed for diagnosing breast cancer patients with optimal interpretability while achieving competitive performance as compared to the state-of-the-art. Alternatively, there are fuzzy systems that have been proposed and applied for multiple medical problems such as SLAVE2 (García et al., 2014), which iteratively learn fuzzy rules of a disjunctive normal searched by a novel genetic algorithm. Although several specialized fuzzy systems have been proposed for various diseases such as Nilashi et al. (2016) and Nilashi et al. (2017), the development of an interpretable fuzzy rule-based system dedicated to dementia diagnosis barely exists in recent literature. As such in response to the desire for an interpretable system for clinical support and the potential of working with tabular data for dementia research, these observations above motivate the underlying research to design an interpretable fuzzy system for dementia diagnosis.

### 2.2. Materials

#### 2.2.1. Participants

The data set under investigation is the popular 'OASIS-2: Longitudinal MRI Data in Nondemented and Demented Older Adults', consisting of a longitudinal collection of 150 subjects aged 60 to 96. These subjects were selected from a larger database of individuals who had participated in MRI studies at Washington University. For each subject, which was scanned on two or more visits, 3 or 4 individual T1-weighted MRI scans obtained in single scan sessions are included, resulting in a total of 373 imaging sessions. The very details of the data set can be found in Marcus et al. (2010).

### 2.3. Pre-processing

A total number of 15 attributes were recorded at every MRI scanning session. In this article, the following 6 attributes are not included to construct the machine learning model with the justifications as follows.

- Subject-ID and MRI-ID are removed for privacy consideration.
- Among the 150 subjects, 72 subjects were identified as “Nondemented” throughout the study; 64 of the subjects were initially marked as “Demented” and remained so for subsequent visits. Another 14 subjects were initially identified as “Nondemented” but were subsequently characterized as “Demented” at a later visit, thus falling under the “Converted” category. With the aim to construct a predictive model for the decision attribute of Clinical Dementia Rating (CDR), the Group attribute, which is directly generated based on the values of CDR (hence highly correlated), is removed to avoid the generation of overly optimistically generated models.
- As each subject was scanned on two or more visits, the “Visit” attribute suggests at which visit the underlying data entry was obtained. Similarly, the “MR Delay,” suggests the number of days between each MRI scan. As both attributes work as a local index and are unlikely to carry any useful information that can be linked to dementia diagnosis, they are also removed.
- The “Hand” attribute is removed, as all subjects are right-handed.

**Figure 1:**
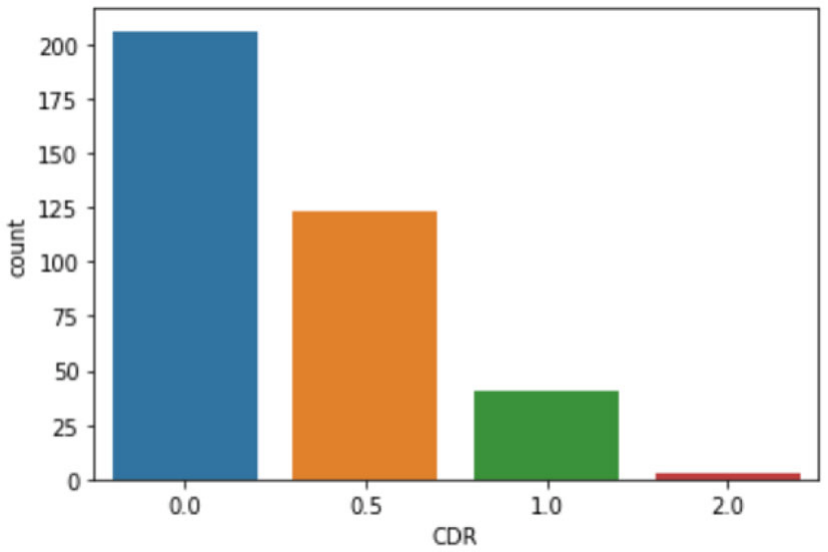
Clinical dementia rating (CDR) distribution.

This results in a data set including measures with associated illustrations as shown in Table 1 (Marcus et al., 2010). Figure 1 demonstrates the distribution of CDR, which suggests the diagnostic outcome. Considering the highly imbalanced diagnostic distribution, subjects with positive diagnostic outcomes are categorized with dementia, as per (Marcus et al., 2010), regardless of their severity. These results in the diagnosis decision variable with 206 subjects without dementia (*CDR* = 0) and 167 subjects with dementia (*CDR* ≥ 0). The baseline performance based on a random guess of the outcome is, therefore, 55.2%. As the range of different attributes varies significantly, a pre-processing step is to normalize each attribute so that their normalized values fall within the range of [0, 1], by updating each original attribute value *x* as $\frac{x-x_{\text{min}}}{x_{\text{max}}-x_{\text{min}}}$, where *x*_max_ and *x*_min_ are the maximum and minimal values of the attribute that *x* belongs to.

**Table 1:** Summary of attributes.

Age	Age at time of image acquisition (years).
Gender	Gender (Male or Female).
EDUC	Years of education.
SES	Socio-economic status labeled into categories from 1 (highest status) to 5 (lowest status).
MMSE	Mini-Mental State Examination score, ranging from 0 (worst) to 30 (best).
eTIV	Atlas scaling factor (unitless). Computed scaling factor that transforms native-space brain and skull to the atlas target (i.e., the determinant of the transform matrix).
nWBV	Estimated total intracranial volume (*cm*^3^).
ASF	Normalized whole brain volume, expressed as a percent of all voxels in the atlas-masked image that are labeled as gray or white matter by the automated tissue segmentation process.
CDR	Clinical Dementia Rating. (0 = no dementia, 0.5 = very mild AD, 1 = mild AD, 2 = moderate AD, 3 = Severe AD).

**Figure 2:**
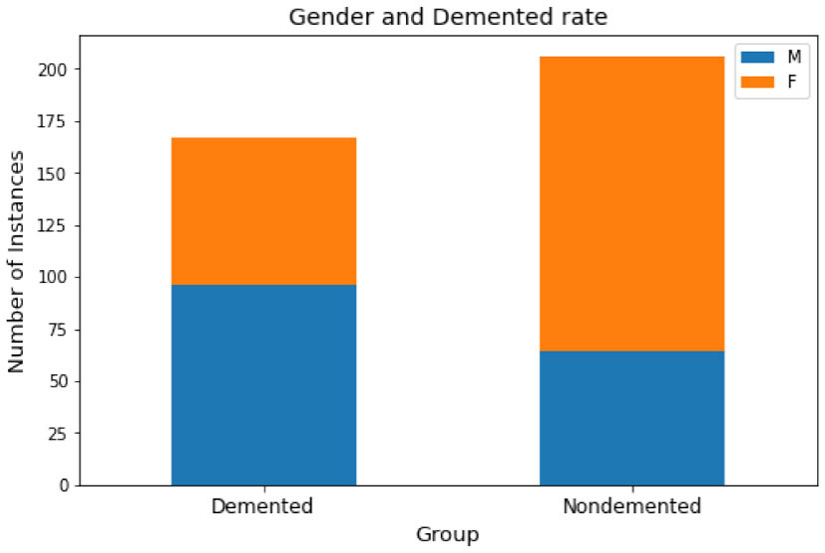
Gender vs. demented.

For the attribute “Gender,” as shown in Figure 2, it is generally equally distributed among the demented group (with *CDR* > 0) but with more women in the non-demented group (with *CDR* = 0). The basic statistics are presented in Table 2. The “Count” measures the number of available records per attribute in the original data, which suggests missing values exist for SES and MMSE. Instead of resorting to more advanced interpolation techniques such as Chen et al. (2019c), this study adopts the traditional data imputation method by filling “SES” and “MMSE” with the corresponding mean value (Belger et al., 2016).

**Table 2:** Statistics of the data set.

**Attribute**	**Count**	**Mean**	**Std**	**Min**	**25*%***	**50*%***	**75*%***	**Max**
Age	373	77.013	7.641	60	71	77	82	98
EDUC	373	14.598	2.876	6	12	15	16	23
SES	354	2.460	1.134	1	2	2	3	5
MMSE	371	27.342	3.683	4	27	29	30	30
eTIV	373	1488.129	176.139	1106	1357	1470	1597	2004
nWBV	373	0.730	0.037	0.644	0.7	0.729	0.756	0.837
ASF	373	1.195	0.138	0.876	1.099	1.194	1.293	1.587
CDR	373	0.291	0.375	0	0	0	0.5	2

**Figure 3:**
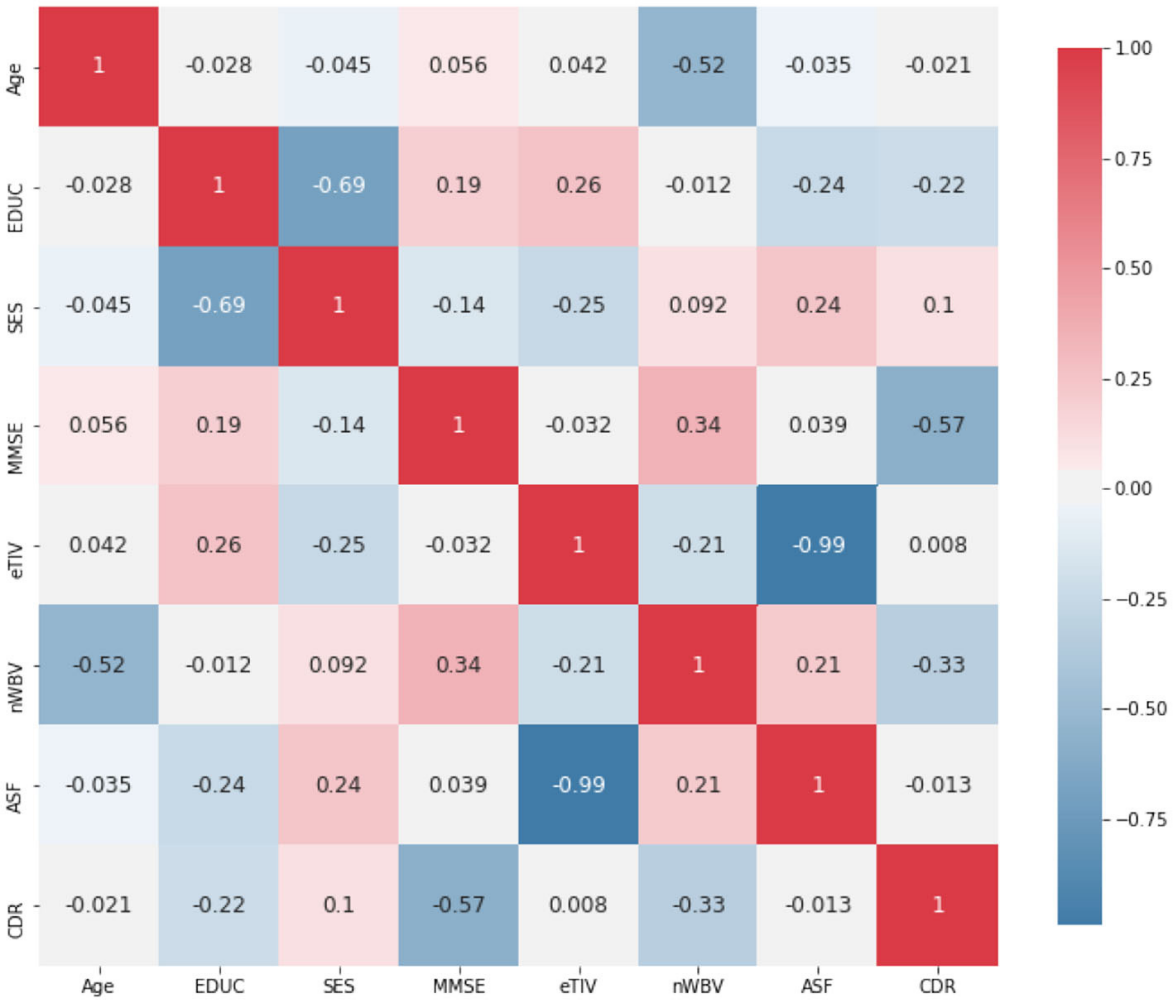
Correlation among attributes.

In addition, Figure 3 demonstrates the Pearson correlation of the attributes in a pair-wise manner, with the value ranging between +1 and −1. A value of +1 is a total positive linear correlation; 0 is no linear correlation; and −1 is a total negative linear correlation. It is not a surprise to see a relatively high correlation between the years of education (EDUC) and socio-economic status (SES). MMSE is a 30-point questionnaire test used extensively in clinical and research settings to measure cognitive impairment, thus not a surprise to see it is relatively highly correlated with CDR. It is also worth noting that the eTIV is very highly correlated on ASF. The eTIV is, therefore, also dropped, since it is automatically derived from and linearly dependent with ASF (Buckner et al., 2004). This leads to a reduced data set with 7 predictors (including Age, Gender, EDUC, SES, MMSE, nWBV, and ASF) for the prediction of CDR.

## 3. Proposed methodology

The key to obtaining an interpretable fuzzy system for dementia diagnosis is to learn a collection of fuzzy if-then production rules that are able to diagnose a subject given the shown symptoms. With no loss of generality, the fuzzy system to learn requires conducting the mapping φ:*X*^*n*^ → *Y*, where *X*^*n*^ is the multidimensional domains for *n* input attributes taken from a subject, and *Y* is the decision variable having *L* possible diagnostic outcomes. As analyzed from the preceding section, this article considers *n* = 7 input attributes (including Age, Gender, EDUC, SES, MMSE, nWBV, and ASF) for the prediction of whether the underlying subject is demented (thus *L* = 2 for the output variable CDR). The behavior of the diagnostic system will be trained following the supervised learning approach, through the collection of *M* = 373 input-output example pairs, where for each observation of the input variables $x_{i}={\left(x_{1}^{1},\ldots ,x_{i}^{k},\ldots ,x_{i}^{n}\right)}^{T},x_{i}^{k}\in X_{i},k=1,2,\ldots ,n,i=1,2,\ldots M$, an associated class *y*_*i*_ ∈ *Y* is indicated.

**Figure 4:**
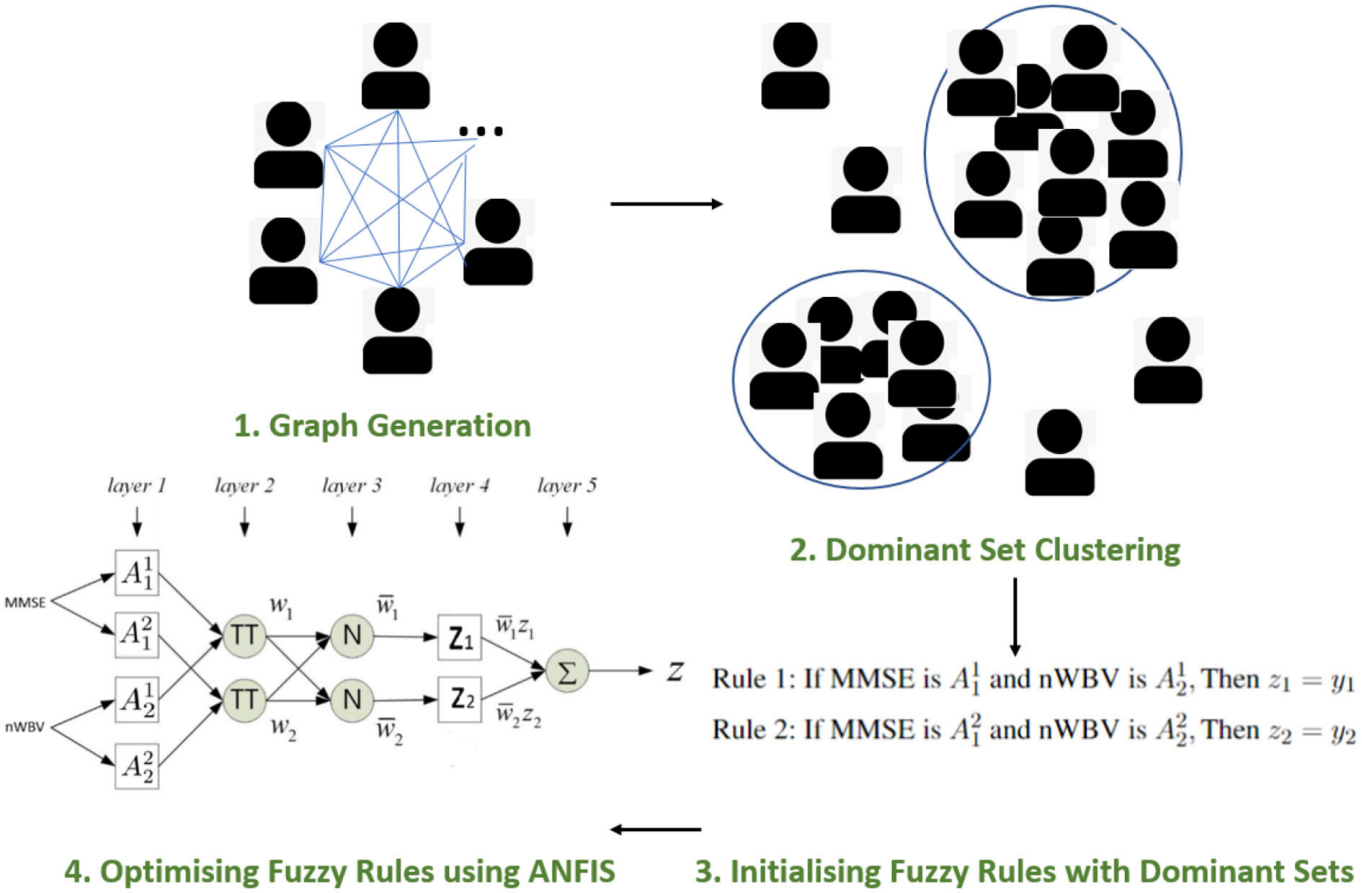
Proposed framework.

To outline the framework of the proposed approach, Figure 4 illustrates the workflow of the model for diagnosing dementia. In particular, The framework starts by constructing an undirected graph, where the verse distance between each pair of subjects represents the edge weight. This is followed by using a peeling-off strategy, iteratively extracting from the graph a dominant set, each representing a cluster of subjects with high mutual similarities and maximality. This sequential search enables the automatic detection of cluster numbers, which are usually manually specified by conventional clustering approaches. Each dominant set is then converted into a parameterized fuzzy rule, and further optimized using the supervised ANFIS neuro-fuzzy framework (Jang, 1993). The following subsections introduce the proposed approach in detail.

### 3.1. Graph generation

The idea of dominant sets originates from the graph theory, by which a continuous formulation of the maximum clique problem is defined in Pavan and Pelillo (2006). Specifically, an undirected graph *G* with weighted edges is represented as *G* = (*V, E*, ω), where *V* represents the set of graph nodes; the edge set *E* ⊆ *V* × *V* includes all possible connections of nodes in the pairwise relationships; ω:*E* → ℝ is a real-valued function which assigns a weight to each edge, reflecting similarity among linked objects.

In the context of dementia diagnosis, an edge-weighted undirected graph can be extracted, where vertices *V* correspond to individual subjects *x*_*i*_ and the edges ω_*ij*_ among the subjects represent the strengths of links between pairs of vertices *x*_*i*_ and *x*_*j*_. As such, a |*V*| × |*V*| symmetric adjacency matrix *A* = {*a*_*ij*_}can be generated to represent such graph. Specifically, the pairwise similarity between subject *x*_*i*_ and *x*_*j*_ is measured by accounting for input attributes values taken using a Gaussian kernel:


(1) $$\begin{array}{l} a_{ij}={𝟙}_{i\neq j}exp\left(-\overset{n}{\underset{k=1}{\sum }}\|x_{i}^{k}-x_{j}^{k}{\|}_{2}^{2}/\sigma^{2}\right) \end{array}$$

where $\overset{n}{\underset{k=1}{\sum }}\|x_{i}^{k}-x_{j}^{k}{\|}_{2}^{2}$ measures the Euclidean distance between subject *x*_*i*_ and *x*_*j*_ over the *n* predictors; σ is defined as the variance of the pairwise distances; 𝟙_*P*_ = 1 if *P* is true, 0 otherwise, which indicates there are no self-loops in the graph with all entries on the main diagonal A being zero.

### 3.2. Dominant set clustering

A dominant set (DS) can then be defined on the basis of similarity values among nodes in *V*. Let *S* ⊆ *V* be a nonempty subset of patient subjects representing the nodes in the graph, *x*_*i*_ ∈ *V* and *x*_*j*_ ∈ *S*. A measure of similarity between *x*_*i*_ and the average similarity of *x*_*j*_ with respect to *x*_*j*_'s neighbors in *S* is defined as:


(2) $$\begin{array}{l} \phi_{S}\left(x_{i},x_{j}\right)=a_{ij}-\frac{1}{\left|S\right|}\underset{x_{k}\in S}{\sum }a_{jk}. \end{array}$$

It can be observed that ϕ_*S*_(*x*_*i*_, *x*_*j*_) can be either positive or negative. The weight of *x*_*i*_ with regard to *S* can be assigned recursively as:


(3) $$\begin{array}{l} W_{S}(x_{i})=\{\begin{array}{ll} 1 & \text{if}|\text{S}|=1\\ \sum_{x_{j}\in S\backslash \{x_{i}\}}\phi_{S\backslash \{x_{i}\}}(x_{j},x_{i})W_{S\backslash \{x_{i}\}}(x_{j}) & \text{otherwise}. \end{array} \end{array}$$

Intuitively, *W*_*S*_(*x*_*i*_) measures the overall similarity between subject *x*_*i*_ and the subjects of *S*\{*x*_*i*_} with respect to the overall similarity among the subjects in *S*\{*x*_*i*_}. A positive *W*_*S*_(*x*_*i*_), therefore, suggests that adding *x*_*i*_ into its neighbors in *S* will increase the internal coherence of the set, whereas a negative value indicates a decreased overall coherence. The total weight of *S* can be computed as:


(4) $$\begin{array}{l} W\left(S\right)=\underset{x_{i}\in S}{\sum }W_{S}\left(x_{i}\right) \end{array}$$

Finally, a non-empty subset of subjects *S* ⊂ *V* such that *W*(*T*) > 0 for any non-empty *T* ⊂ *S*, is said to be a dominant set if:


(5) $$\begin{array}{l} W_{S}\left(x_{i}\right)>0,\text{for all }x_{i}\in S, \end{array}$$

(6) $$\begin{array}{l} W_{S\cup \left\{x_{i}\right\}}\left(x_{i}\right)<0,\text{for all }x_{i}\notin S. \end{array}$$

Dominant sets can then be identified by the solutions to the linearly-constrained quadratic problem:


(7) $$\begin{array}{l} \begin{array}{cc} \text{maximize} & f\left(\text{z}\right)={\text{z}}^{⊤}A\text{z}\\ \text{subject to} & \text{z}\in \Delta \end{array} \end{array}$$

where


$$\begin{array}{l} \Delta =\;\left\{\text{z}\in R^{M}:\overset{M}{\underset{i=1}{\sum^{\text{​}}}}z_{i}=1\text{ and }z_{i}\geq 0\text{ for all }i=1,\cdots ,M\right\}. \end{array}$$

A strict local solution z^*^ of Equation (7) is the named weighted characteristic vector, where *z*_*i*_ > 0 suggests that the node *x*_*i*_ in question is in a dominant set of *G*, and **z**^⊤^ is the transpose of **z**. An effective optimization approach for solving Equation (7) is given by the so-called *replicator dynamics*:


(8) $$\begin{array}{l} z_{i}^{\left(t+1\right)}=z_{i}^{\left(t\right)}\frac{{\left(A{\text{z}}^{\left(t\right)}\right)}_{i}}{{{\text{z}}^{\left(t\right)}}^{⊤}A{\text{z}}^{\left(t\right)}}, \end{array}$$

where *i* = 1, 2, ⋯   , *M*. It has been proven that for any initialization of **z** ∈ Δ, its trajectory will remain in Δ with the increase of iteration *t*. With the increasing of *t* in Equation (8), the objective function *f*(**z**) in Equation (7) is either strictly increasing or remains a constant. In practice, the stopping criteria of the dynamic system can be set as a maximal number of iteration *t* or a minimal increment of *f*(**z**).

### 3.3. Initializing fuzzy rules with dominant sets

For the solution of replicator dynamics, A peeling-off strategy is adopted by Pavan and Pelillo (2006), which iteratively extracts a subset of subjects belonging to the same branch, i.e., a dominant set *S*, each time by using Equation (8) and repeats the process in the new set of nodes *V* = *V*\*S*. Within the framework of the dominant set, a dominant set enables to represent a cluster, for it identifies a subset of objects satisfying two basic properties of a cluster, i.e., the internal homogeneity requesting that elements belonging to the cluster should have high mutual similarities, and maximality indicating a cluster cannot be further extended by introducing external elements.

The dominant sets-inspired clusters thus empower the identification of major patterns arising from the input data, which can then be converted into parameterized fuzzy rules for further optimization. Note that different from traditional clustering approaches that insist on partitioning all subjects, and hence subjects that are not similar enough may be forced to be put into coherent groups, the dominant set-based approach considers the clustering as a sequential search of structures, which enables to keep unstructured and diverse clutter. In addition, the sequential search by following the peel-off strategy empowers the automatic identification of cluster numbers over that typically pre-determined by conventional clustering approaches such as k-means.

Assume a cohort of dominant sets *S*_1_, …, *S*_*d*_…, and *S*_*D*_ is extracted by the peel-off approach. Each dominant set can be deemed as a special group that reflects characteristics bounded by subjects from the underlying cohort. Thus, it is natural to convert such dominant set/cluster *S*_*d*_ into a corresponding fuzzy rule *R*_*d*_ in the form of:


(9) $$\begin{array}{l} \text{If}x^{1}\text{ is}A_{1}^{d}\text{ and }\ldots \text{ and }x^{n}\text{ is }A_{n}^{d},\text{Then}z_{d} \end{array}$$

where *d* = 1, 2, …, *D*, with *D* representing the total number of rules in the fuzzy system; *x*^*k*^, *k* = 1, …, *n* is the *k*-th domain variable and takes values from *X*_*k*_ with $A_{k}^{d}$ denoting a fuzzy set it may take; and *z*_*d*_ is the rule consequent that describes the diagnosis.

Given a collection of subjects $x_{1}^{\left(d\right)},\ldots ,x_{i}^{\left(d\right)},\ldots ,x_{\left|S_{d}\right|}^{\left(d\right)},$ assigned to the exclusive cluster *S*_*d*_, where |*S*_*d*_| represents the size or the total number of subjects included in this cluster, the specification of fuzzy set $A_{k}^{d}$ for input attribute *x*^*k*^ can be implemented as follows. Although the specific membership function used for a fuzzy set may be better considered as a result of the consultation with clinical experts, this paper empirically considers the use of popular Gaussian membership functions as:


(10) $$\begin{array}{l} \mu_{A_{k}^{d}}\left(x^{k}\right)=exp\left(-{\left(\frac{x^{k}-c_{d}^{k}}{\sigma_{d}^{k}}\right)}^{2}\right) \end{array}$$

where $c_{d}^{k}$ denotes the mean value and $\sigma_{d}^{k}$ represents standard deviation. Specifically, the mean value is initialized using the average of subjects values projected into the corresponding dimension as $x_{i}^{\left(d\right)}$, such that


(11) $$\begin{array}{l} c_{d}^{k}=\frac{\sum_{x_{i}\in S_{d}}x_{i}^{k}}{\left|\left\{x_{i}\in S_{d}\right\}\right|} \end{array}$$

Similarly, the $\sigma_{d}^{k}$ is computed by


(12) $$\begin{array}{l} \sigma_{d}^{k}=\sqrt{\frac{\sum_{x_{i}\in S_{d}}{\left(x_{i}^{k}-c_{d}^{k}\right)}^{2}}{\left|\left\{x_{i}\in S_{d}\right\}\right|}} \end{array}$$

The connection between individual logical predicates (e.g., $x^{1}\text{is}A_{d1}$) is then implemented using the product T-norm as *T*_prod_(*a, b*) = *a*·*b*, where *a* and *b* represent the truth values of two logical statements. The consequence of the fuzzy rule can then be specified by counting the majority of the class labels of the responding subjects delimited by the underlying cluster. As a result, the fuzzy rule mapped from an original dominant set-inspired cluster *S*_*d*_ can be represented as


(13) $$\begin{array}{l} \begin{array}{c} \text{If}x^{1}\text{is}e^{-{\left(\frac{x-c_{d}^{1}}{\sigma_{d}^{1}}\right)}^{2}}\text{and }\ldots \text{ and}x^{n}\text{is}e^{-{\left(\frac{x-c_{d}^{n}}{\sigma_{d}^{n}}\right)}^{2}},\text{then}z_{d}=y_{d} \end{array} \end{array}$$

where $e^{-{\left(\frac{x-c_{d}^{i}}{\sigma_{d}^{i}}\right)}^{2}}$ is the Gaussian membership function for attribute *x*^*i*^ with $c_{d}^{i}$ and $\sigma_{d}^{i}$ computed as above, and *y*_*d*_ represents the decision label determined as the majority class of the corresponding cluster. It is worth noting that apart from being derivable in its whole domain, the Gaussian membership function may be interpreted as, e.g., 'MMSE is Around 26' when its mean is 26 for MMSE, which eases its communication to non-expert users in clinical practice.

### 3.4. Optimizing converted fuzzy rules using ANFIS

The use of fuzzy rules comprised of fuzzy sets and fuzzy logical operators offers an effective approach to dealing with uncertainty and impression that commonly exist in medical data. Moreover, a fuzzy rule-based system supports approximate reasoning, which is closer to human reasoning and aims to generate an inexact conclusion from inexact premises. However, a direct transformation of dominant sets into fuzzy rules is unlikely to generate accurate diagnoses for dementia, as the rules directly resulting from clusters of unsupervised nature may only identify rough input patterns without explicitly utilizing the labeled outputs. As such, a supervised learning procedure is required to fine-tune the parameters embedded in the preliminary fuzzy system to best approximate the link between input and output patterns in the data.

Particularly, the adaptive network-based fuzzy inference system (ANFIS) (Jang, 1993) is utilized for subsequent optimization, which is a popular Takagi-Sugeno-Kang (TSK) fuzzy inference system that combines the parallel computation and learning abilities of neural networks supported with the knowledge representation and reasoning abilities of fuzzy systems. The ANFIS structure can now be initialized with previously generated fuzzy rules to be optimized further by adapting associated fuzzy rule parameters for improved performance.

**Figure 5:**
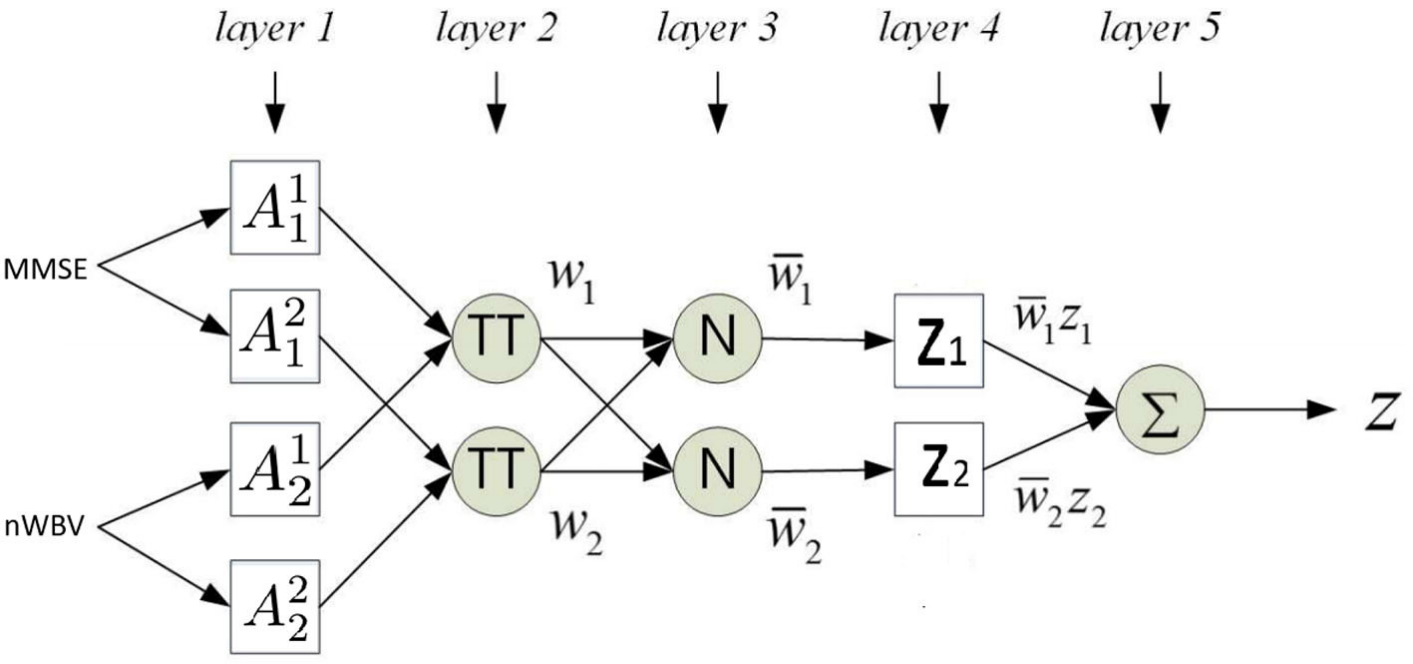
Adaptive network-based fuzzy inference system (ANFIS) framework.

The section under the framework of ANFIS discusses the parameters to optimize and how the inference of approximate reasoning is performed to reach an overall diagnosis through matching individual fuzzy rules. To ease the illustration, only the two most significant features are used, i.e., MMSE and nWBV, which have the greatest correlation with the diagnosis as shown in Figure 3. Suppose only two fuzzy rules learnt by the dominant set-based clustering for a simplified illustration, which are into the following TSK fuzzy rules:


(14) $$\begin{array}{l} \begin{array}{c} \text{Rule }1:\text{ If MMSE is }A_{1}^{1}\text{ and nWBV is }A_{2}^{1},\text{ Then }z_{1}=y_{1}\\ \text{Rule }2:\text{ If MMSE is }A_{1}^{2}\text{and nWBV is}A_{2}^{2},\text{ Then }z_{2}=y_{2} \end{array} \end{array}$$

where $A_{1}^{1}$ and $A_{2}^{1}$ are fuzzy sets for variable MMSE and nWBV in Rule 1, and $A_{1}^{2}$ and $A_{2}^{2}$ for Rule 2. The flat fuzzy rule base consisting of the two above rules can be converted under the neuro-fuzzy ANFIS framework as shown in Figure 5, where parameters embedded in the square nodes are allowed to adapt, whereas the circle nodes remained fixed throughout the learning process. The workings of individual ANFIS layers are detailed below.

***Layer 1*** contains several square nodes—each defines a membership function $\mu_{A_{k}^{d}}\left(x^{k}\right)$ of variable *x*^*k*^ for the *d*-th rule. For instance, $A_{1}^{2}$ is the membership function of the first variable (MMSE in this case) for second rule. The specification of each membership function involves the computation of mean and SD, which follows as per Equations 11 and 12. These premise parameters are subject to be adjusted in subsequent learning processes.

***Layer 2*** contains several circle nodes—each accumulates the incoming firing degrees through the product logical operator. The firing strength *w*_*d*_ for Rule *d, d* = 1, 2 can thus be represented as:


(15) $$\begin{array}{l} O_{d}^{2}=w_{d}=\mu_{A_{1}^{d}}\left(x^{1}\right)\times \mu_{A_{2}^{d}}\left(x^{2}\right) \end{array}$$

***Layer 3*** contains several circle nodes—each computes the normalized contribution of the *i*th rule's firing strength over the total contributions made by all rules:


(16) $$\begin{array}{l} O_{d}^{3}=\bar{w_{d}}=\frac{w_{d}}{\sum_{d=1}^{D}w_{i}} \end{array}$$

where *D* represents the total number of rules available (*D* = 2 for the present example).

***Layer 4*** contains square nodes, each computed with the following function:


(17) $$\begin{array}{l} O_{d}^{4}=\bar{w_{d}}z_{d}=\bar{w_{d}}\left(y_{d}\right) \end{array}$$

where *y*_*d*_ is a polynomial of input values and contains parameters to be optimized further (termed the consequent parameters). Note the choice of a zero-order rule consequent in this article makes it possible to interpret the classification for clinical decision support, whereas commonly used first order TSK rules such as $z_{d}=p_{d}x^{1}+q_{d}x^{2}+y_{d}$ where *p*_*d*_ and *q*_*d*_ are additional parameters, suit better for regression problems.

***Layer 5*** contains a single circle node in this output layer that calculates the overall output given the underlying instance as


(18) $$\begin{array}{l} O_{1}^{5}=\underset{d}{\sum }\bar{w_{d}}z_{d}=z \end{array}$$

The above details the computation of how an instance is mapped against the ANFIS layers. Parameters embedded in the structure, including the premise and consequent parameters, are then tuned through a hybrid optimization method. In each training epoch, consequent parameters are optimized using the least squares estimation method in a forward pass; while premise parameters are determined using gradient descent in a backward pass. The details of such computation are beyond the article's scope but can be identified in Jang (1993).

## 4. Experimentation and discussions

The experimentation and discussions of the proposed approach are provided in this section, including the demonstration of an illustrative example, as well as further studies compared with alternative popular machine learning techniques.

### 4.1. Illustrative example

This subsection demonstrates how the proposed approach may be employed to effectively supporting in clinical decision making. For the sake of visualization, only the two most significant features as calculated in Figure 3 are used, i.e., the MMSE and the nWBV. It is worth noting that all seven attributes introduced in Section 2.2 have been used to construct the models for systematic evaluations to present in Section 4.2.

**Figure 6:**
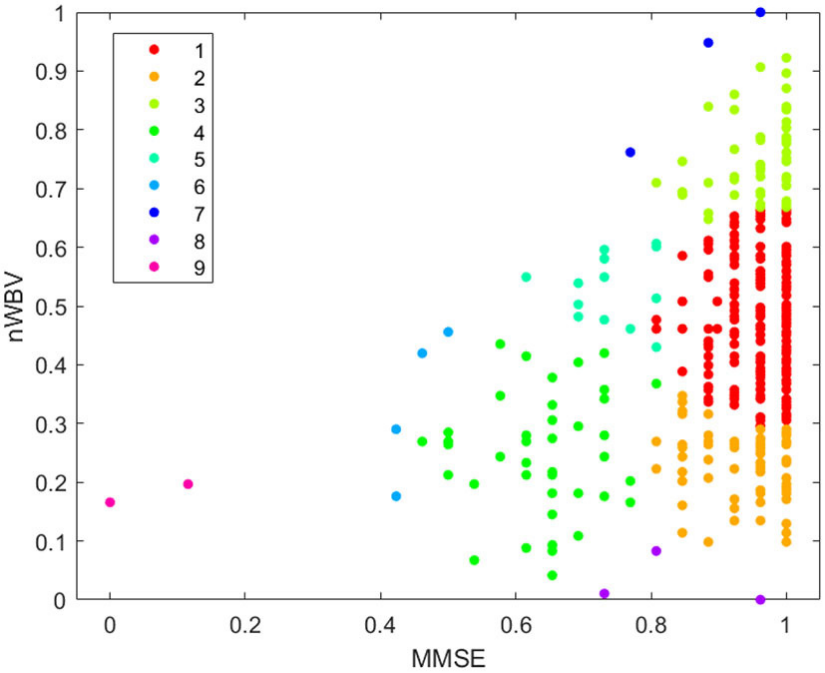
Clustering by dominant sets.

In order for the DS-based clustering algorithm to work, the similarity between pairs of instances is calculated as per Equation 1. The peeling-off approach is then used to iteratively extract a cluster of instances that possess the internal homogeneity and maximality as per described in Section III-B. As a result, nine clusters are generated as shown in Figure 6. Different from traditional clustering approaches that are based on the idea of partitioning the input data into a predetermined number of classes, one of the biggest advantages of using the DS-based approach is that it enables to automatically detect unstructured and diverse clutter such as the 9-th cluster in Figure 6, while also empowering the automatic determination of clustering number. Whereas, traditional clustering approaches based on the idea of partitioning the input data into a predetermined number of classes could potentially force to merge the two noisy points in cluster 9 with the main cluster, especially when the predetermined cluster number is inappropriately specified, which is practically difficult when working with high dimensional data.

**Figure 7:**
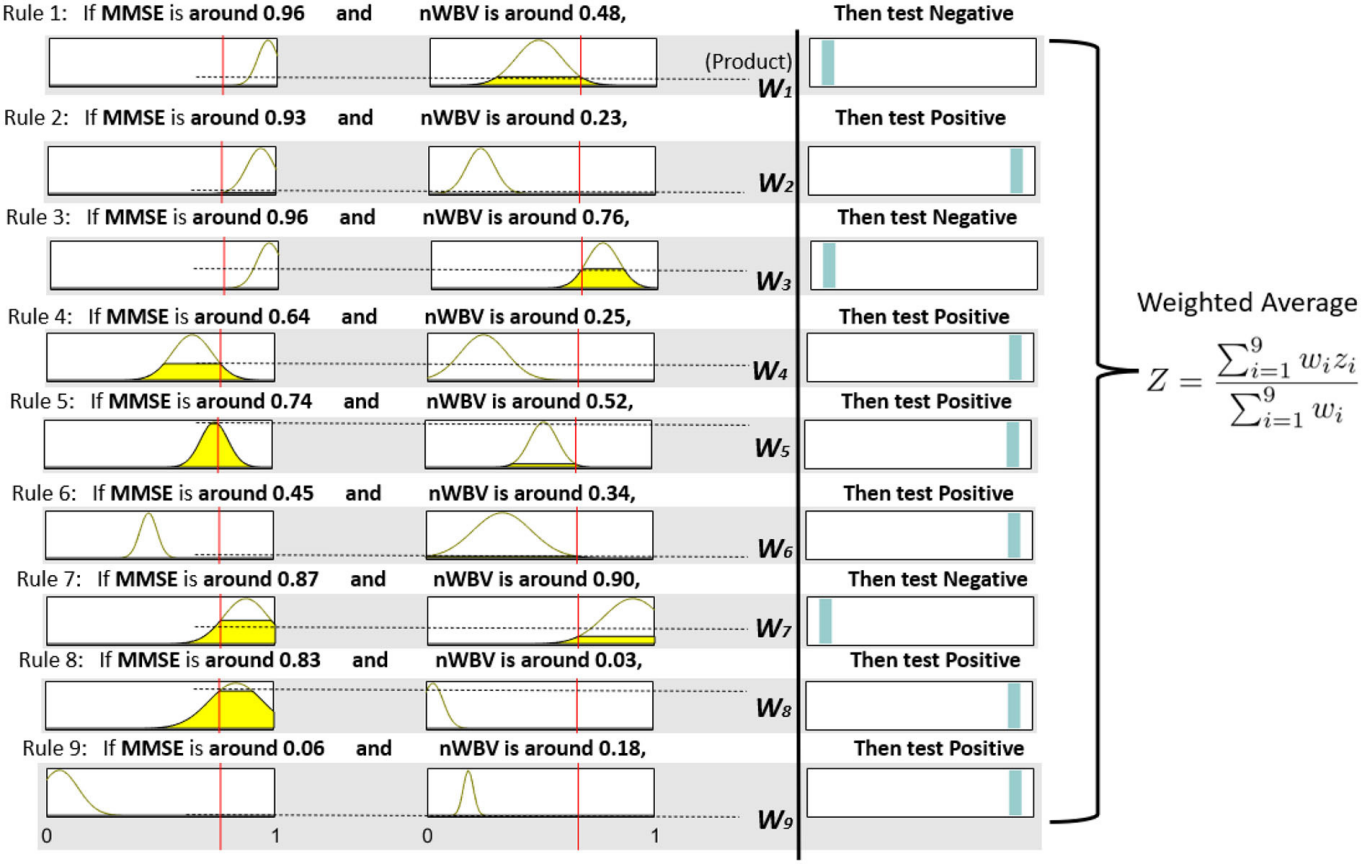
Fuzzy rule base and inference process.

Each extracted cluster is then converted into a fuzzy rule. This is executed by first projecting instances within the cluster into each dimension axis and computing the mean and SDs to initialize the Gaussian membership functions as per Equation (11) and (12). The rule consequent is then determined by voting the majority of class labels. A rule base consisting of nine fuzzy rules can be represented in Figure 7, which demonstrates each rule in fuzzy (in the form of Gaussian membership functions) and linguistic (in the form of fuzzy numbers) terms. For instance, Rule 1, which should have been as follows in fuzzy terms,

- If MMSE is $e^{-\frac{{\left(x-0.96\right)}^{2}}{2*0.04^{2}}}$ and nWVB is $e^{-\frac{{\left(x-0.48\right)}^{2}}{2*0.10^{2}}}$, Then test *Negative*.

can be simplified by converting the associated Gaussian membership function into a center-based fuzzy number (in this case, “MMSE is $e^{-\frac{{\left(x-0.96\right)}^{2}}{2*0.04^{2}}}$” can be communicated as “when MMSE is around 0.96” to facilitate its communication in clinical practice). Note the domain of each feature space has been normalized in the unit interval to enhance model generalization capability that might be affected by features with large domains.

**Figure 8:**
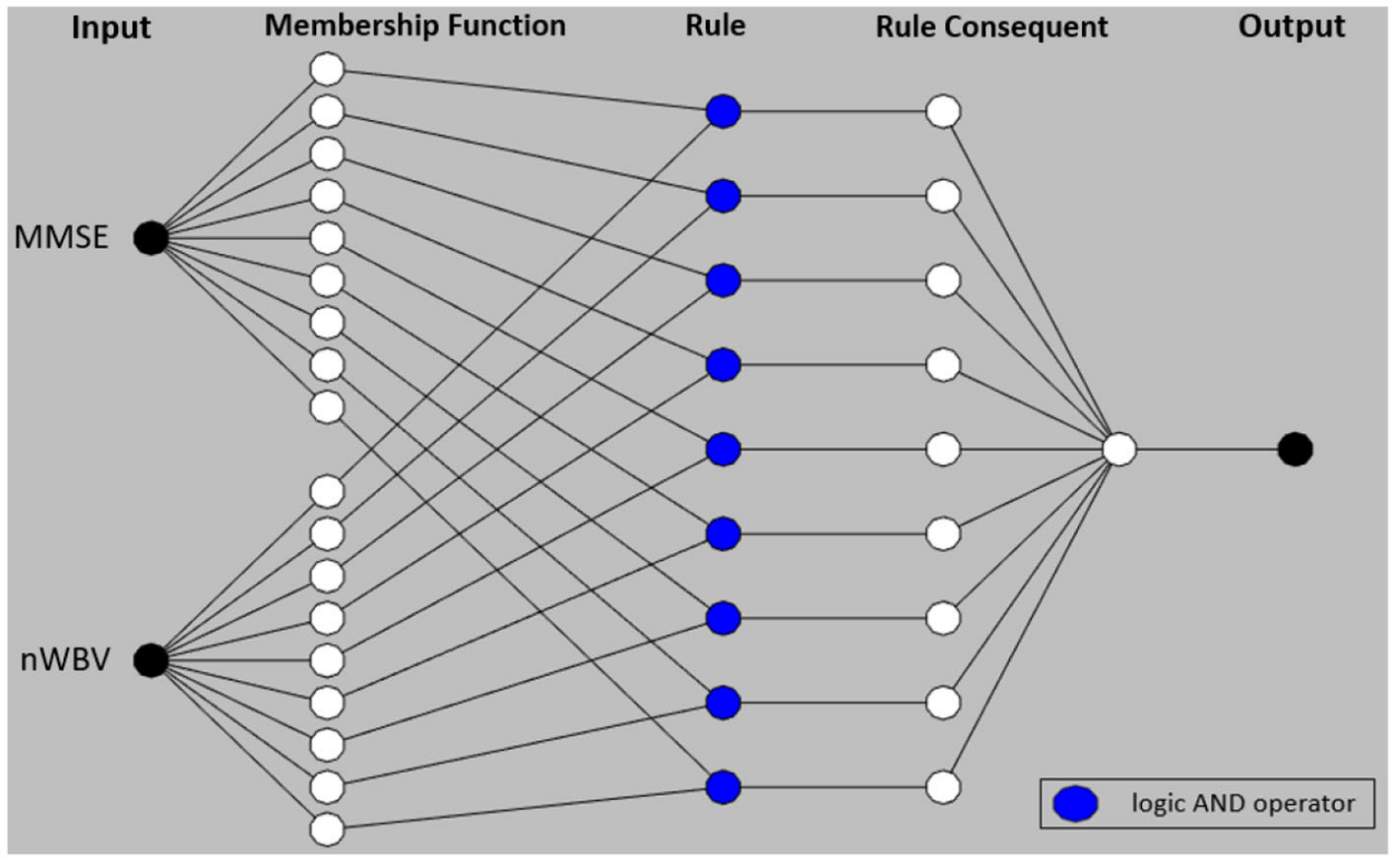
Adaptive network-based fuzzy inference system structure.

The neuro-fuzzy ANFIS framework is then utilised to optimize previously converted fuzzy rule base by fine-tuning both antecedent and consequent parameters in Figure 8. To demonstrate how reasoning is conducted through the obtained fuzzy rule base for aiding clinicians to reach a diagnosis, consider a patient of the following test results, i.e., (MMSE = 24, nWBV = 0.771), which are then normalized as (MMSE≈0.761, nWBV≈0.664) as shown with the red lines in Figure 7. The proposed approach starts by computing the matching degree of each available crisp test result against the membership functions of the associated fuzzy sets for the underlying variable. This can be visualized to demonstrate the level of fulfillment through the yellow patches in the Gaussian membership functions, which are annotated using linguistic fuzzy numbers in the example, to support the communication and understanding of derived knowledge to non-fuzzy experts such as clinicians and patients.

Once the firing degrees of the membership functions are calculated with respect to the corresponding antecedent variables for a given rule, the overall matching degree with respect to all rule condition variables is computed by applying the associated fuzzy operator (i.e., the product operation in this example). The resultant activation strength of a given rule is then normalized to calculate its contribution relative to those from alternative rules in the rule base. The consequents of individual rules weighted by the associated normalized activation degrees are finally averaged to produce the overall output of the entire reasoning process. In this example, the overall outcome for the given instance is 0.776, which can be rounded to 1, suggesting a positive diagnosis, along with a confidence of 77.6%.

**Figure 9:**
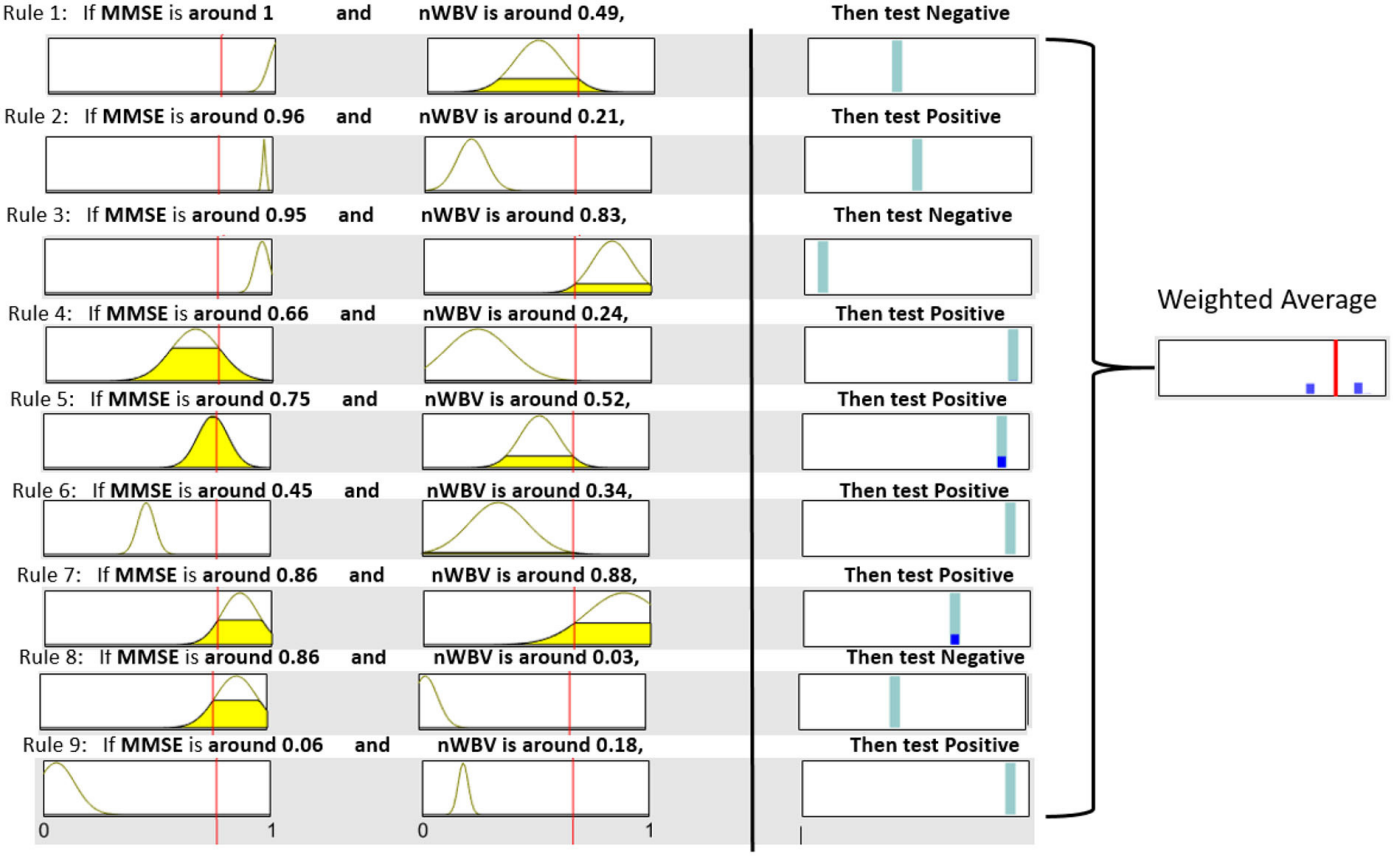
Resultant rule base after ANFIS optimization.

To demonstrate the effect of ANFIS optimization, Figure 9 shows the resultant rule base. From a holistic perspective, the number of rules with the selection of associated variables remains identical, which is expected as ANFIS only optimizes parameters embedded in rule antecedents and consequents without touching the rule base structure. For rule antecedents parameters, numerous adjustments of the parameterized centers and standard deviations of the Gaussian membership functions can be observed, but with most being non-dramatic except the two for MMSE in Rule 1 and 2 that have clearly been shrunk and moved after optimization. The ANFIS has also optimized rule consequents that can be interpreted as rule confidence levels through the position of the cyan bars, which can be clearly observed in Rule 1,2,7, and 8. These changes have collectively resulted in a more powerful diagnostic model that better characterizes patterns exhibited in the underlying patient group, as to be systematically examined in the next subsection.

It is also worth noting that instead of calculating the exact confidence, the weighted average of firing degrees across all rule consequents can also be visualized through the blue bars that represent the firing degrees of associated rules in Figure 9. In this example with regard to the same patient used above, only Rule 5 and 7 are fired to the level as shown by corresponding blue bars. These resulted in an overall weighted average encoded as the red bar that represents the final decision. The decision confidence can be visualized through the relative position of the red bar to the two outmost points, representing a clear negative and positive diagnosis, respectively. As such, this example suggests a positive diagnosis, which the red bar is much closer to.

In summary, the given example demonstrates not only an interpretable system, which delivers human-readable rules but also supports explaining the overall decision by decomposing it into component decisions made by individual rules through a robust inference framework. Furthermore, the fuzzy rule-based system supports approximate reasoning that allows to partially match the given symptoms with multiple rules to varying degrees. This enhances the toleration level of any crisp rule-based system, which either matches inputs to full or none and may, thus, be sensitive to noisy outliers commonly arising from clinical data. This is further supported by an illustrative demonstration such as Figure 9 that could clearly demonstrate the level of fulfillment and decision preference through highlighted patches and bars while omitting as much mathematics as possible, hence further enhancing the delivery of the extracted knowledge to lay users.

### 4.2. Systematic evaluation

In order to systematically assess the performance of the proposed system, the stratified 10-fold cross-validation (10-CV) is employed, where the full OASIS-2 data set is partitioned into ten subsets, with each subset used in turn to assess the performance of the fuzzy system trained on the remaining nine subsets. This 10-CV is then repeated 10 random times with results averaged to generate the experimental results below.

Table 3 explores the effect of fine-tuning using the supervised ANFIS framework, where the DS column specifies the performance of fuzzy rules directly extracted from dominant set-based clustering and DS-ANFIS presents the fuzzy rules optimized by ANFIS. Note the results shown in each run are on the basis of the same random seed, suggesting both DS-initialized fuzzy rules are exactly identical. As shown in Table 3, the SDs within each run are generally much higher than those averaged over the 10 whole runs. This is expected as the performance variations among each fold of the 10-CV tend to be large with the selection of different training and testing data, whereas the variations of the averaged accuracies across different runs tend to be small.

**Table 3:** Effect of parameter tuning with classification accuracy (%).

**Run**	**DS-ANFIS**	**DS**	**#Rules**
1	**82.01 ± 6.92**	68.83 ± 9.72	13.6
2	**81.77 ± 6.71**	67.01 ± 7.19	13.8
3	**81.77 ± 6.50**	69.41 ± 8.20	13.9
4	**80.46 ± 6.72**	68.64 ± 7.15	14.1
5	**81.23 ± 5.68**	65.38 ± 10.40	14.1
6	**81.49 ± 7.66**	66.49 ± 9.92	14
7	**80.43 ± 7.27**	67.06 ± 6.34	13.7
8	**81.79 ± 3.66**	64.27 ± 9.32	13.9
9	**81.79 ± 6.85**	65.92 ± 8.02	14.4
10	**84.21 ± 4.99**	69.72 ± 4.62	14.2
Average	**81.694 ± 1.05**	67.274 ± 1.83	13.97

It is not surprising that DS-ANFIS exhibits better performance across all random runs, owing to the learning of rule parameters in a supervised manner. Overall, DS-ANFIS clearly outperforms DS with a margin of over 14%, which demonstrates the necessity to fine-tune the initialized converted fuzzy rules in a supervised manner. The last column of Table 3 demonstrates the number of dominant sets extracted, thus the cardinality of the resultant fuzzy system. With fewer than 14 rules on average, this implies the size of the fuzzy rule base resulting from the automatic determination of DS-based clustering is compact, which makes it possible to interpret by human experts (Chen et al., 2018).

**Figure 10:**
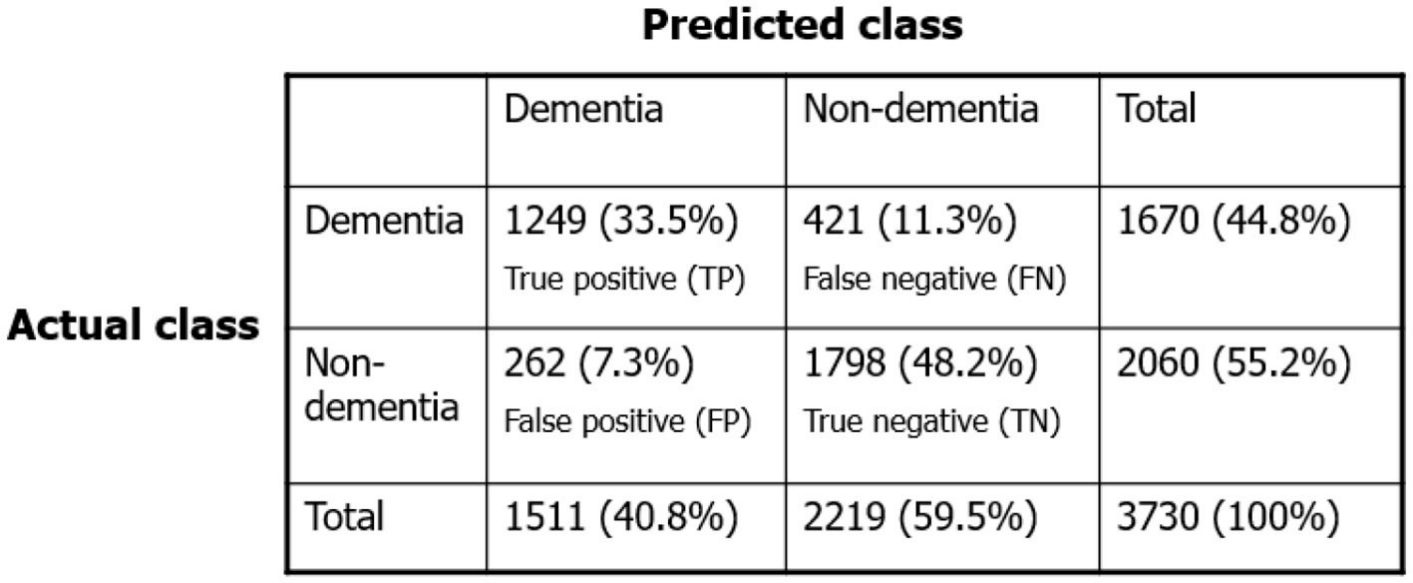
Contingency table/confusion matrix.

Figure 10 presents the full results after performing 10 random times of a 10-CV, resulting in a total number of 3,730 instances in the contingency table/confusion matrix. Both the number of observations and the percentage of the total number of observations are shown in each cell. The rows correspond to the actual diagnosis (ground truth) and the columns correspond to the predicted diagnosis by the proposed approach. The first two diagonal cells show the number and percentage of correct classifications by DS-ANFIS. As such, 1,798 instances are correctly classified as non-demented, corresponding to 48.2% of all 3,730 instances; while 1,249 cases are correctly classified as demented, corresponding to 33.5% of all instances. Conversely, 11.3% of demented instances on the off-diagnosis are incorrectly categorized as non-demented; while 7.0% of normal cases are incorrectly classified as demented. Overall, these result in the precision = $\frac{TP}{TP+FP}=82.7\%$; recall = $\frac{TP}{TP+FN}=74.7\%$; specificity = $\frac{TN}{TN+FP}=87.3\%$.

To evaluate further the performance of the proposed approach, this article compares several popular (non-fuzzy) machine learning and fuzzy rule-based classifiers, which are summarized as follows.

**J48** is a JAVA implementation of the popular C4.5 decision tree algorithm. It has been described as “a landmark decision tree program that is probably the machine learning workhorse most widely used in practice to date" (Witten et al., 2016) and ranked as #1 in the Top 10 Algorithms in Data Mining eminent article by Wu et al. (2008).

**SVM** (Shawe-Taylor and Sun, 2011) has a sound theoretical foundation based on the statistical learning framework, requires only a dozen examples for training, and is insensitive to the number of dimensions (Wu et al., 2008). It has been noted as the most commonly applied method for dementia diagnosis in several recent surveys (Pellegrini et al., 2018; Tanveer et al., 2020).

**RandomForest** (Verikas et al., 2011) is a powerful ensemble learning method for predictive modeling that operates by constructing a multitude of decision trees, whose predictions are then combined through a majority vote to reach an overall decision. Random Forest has also been commonly used for a large variety of tasks including dementia diagnosis (Pellegrini et al., 2018; Tanveer et al., 2020).

**SGERD** (Mansoori et al., 2008) extract a compact set of fuzzy rules based on a novel steady-state genetic algorithm, in which a non-random selection mechanism supports that only the best individuals can survive, with an enhancing function to further assess the candidate rules more effectively before selection.

**SLAVE2** (García et al., 2014) learns fuzzy rules of a disjunctive normal form in an iterative manner. As an improved version of SLAVE (Gonzblez and Pérez, 1999), SLAVE2 proposed novel calculus of the positive and negative examples, fitness functions, and genetic operators to support the identification of individuals' fuzzy rules.

**QuickRules** (Riza et al., 2014) is a novel hybrid approach for fuzzy-rough set rule induction. By performing feature selection and rule induction simultaneously, the generated rule sets are guaranteed to be compact and transparent.

As reviewed in Sections I and II, J48, SVM, and RandomForest have been extensively used in numerous works for dementia diagnosis, while SGERD, SLAVE2, and QuickRules commonly serve as benchmark fuzzy systems in various domains including healthcare. Note the implementations of J48, SVM, RandomForest, and QuickRules can be found in WEKA software (Witten et al., 2016), while those of SGERD and SLAVE2 can be found in the KEEL software (Alcalá et al., 2010), all with default parameter settings.

Table 4 shows the performances of 10 random runs in terms of accuracy. Overall, the proposed DS-ANFIS and RandomForest are the only algorithms with over 80% accuracies. Supported with the two-tale paired *t*-test at the significance level of 0.01, the proposed method statistically beats SVM, the most popular classifier in dementia diagnosis, as well as J48 and the three recent fuzzy rule-based methods. In spite of slightly better overall accuracy, it is statistically equivalent to the powerful random forest method.

**Table 4:** Comparison of classification accuracy (%) against alternative approaches, with v, -, and * suggesting statistically better, same, and worse performance than the proposed work at *p* < 0.01.

**Run**	**DS-ANFIS**	**J48**	**SVM**	**RandomForest**	**SGERD**	**SLAVE2**	**QuickRules**
1	**82.01 ± 6.92**	79.12 ± 6.87	78.58 ± 7.85	80.17 ± 4.88	71.63 ± 5.38	79.61 ± 4.10	76.12 ± 8.45
2	**81.77 ± 6.71**	78.05 ± 5.29	77.48 ± 4.08	80.45 ± 3.91	75.85 ± 6.22	78.80 ± 6.40	77.20 ± 4.79
3	**81.77 ± 6.50**	78.26 ± 6.82	78.27 ± 6.65	80.95 ± 4.16	75.59 ± 5.06	78.26 ± 4.74	79.37 ± 8.46
4	80.46 ± 6.72	77.99 ± 4.47	77.20 ± 4.86	**82.53 ± 7.20**	77.21 ± 4.39	78.56 ± 5.63	78.77 ± 7.73
5	**81.23 ± 5.68**	78.02 ± 4.97	77.43 ± 6.11	80.94 ± 7.94	76.38 ± 8.32	79.65 ± 6.00	78.55 ± 7.54
6	**81.49 ± 7.66**	77.97 ± 6.31	76.93 ± 4.28	81.47 ± 6.87	76.44 ± 7.62	76.64 ± 7.43	77.16 ± 7.58
7	80.43 ± 7.27	77.25 ± 7.64	78.28 ± 4.85	79.64 ± 4.35	74.77 ± 10.91	**80.72 ± 3.82**	78.85 ± 5.80
8	81.79 ± 3.66	76.63 ± 5.56	79.04 ± 7.04	**82.28 ± 6.69**	72.99 ± 8.22	79.90 ± 4.68	80.93 ± 6.98
9	81.79 ± 6.85	78.02 ± 7.79	77.77 ± 6.75	**82.02 ± 6.78**	72.61 ± 9.05	79.62 ± 6.75	79.04 ± 7.51
10	**84.21 ± 4.99**	77.45 ± 7.97	78.02 ± 4.68	83.09 ± 6.27	73.98 ± 8.57	80.11 ± 5.53	80.17 ± 7.17
Average	**81.694 ± 1.05**	77.876 ± 0.66	77.900 ± 0.66	81.354 ± 1.12	74.744 ± 1.87	79.186 ± 1.16	78.616 ± 1.45
Ttest *p*-value		4.82E-06 (*)	1.77E-06 (*)	0.3594 (-)	1.00E-05 (*)	3.89E-04 (*)	1.66E-04 (*)

However, the pairwise *t*-test procedure is not able to derive an overall conclusion involving more than one pairwise comparison, as the error will be accumulated from its combinations, which is the family-wise error rate, defined as the probability of making one or more false discoveries among all the hypotheses when performing multiple pairwise tests (Demšar, 2006). To further validate the overall superior performance that DS-ANFIS possesses over its alternatives, non-parametric statistical tests are also employed here. In particular, the Friedman test (Demšar, 2006) (Friedman two-way analysis of variances by ranks) is applied to detect whether there is indeed any statistically significant difference among the seven algorithms as a group. The Friedman test applies when the number of datasets *n* or the number of classifiers for comparison *k* is large, i.e., *n* > 15 or *k* > 4 - in our case, although we only have 10 sampled sets through cross-validation, the 6 alternative classifiers used for comparison make the Friedman test appropriate.

**Figure 11:**
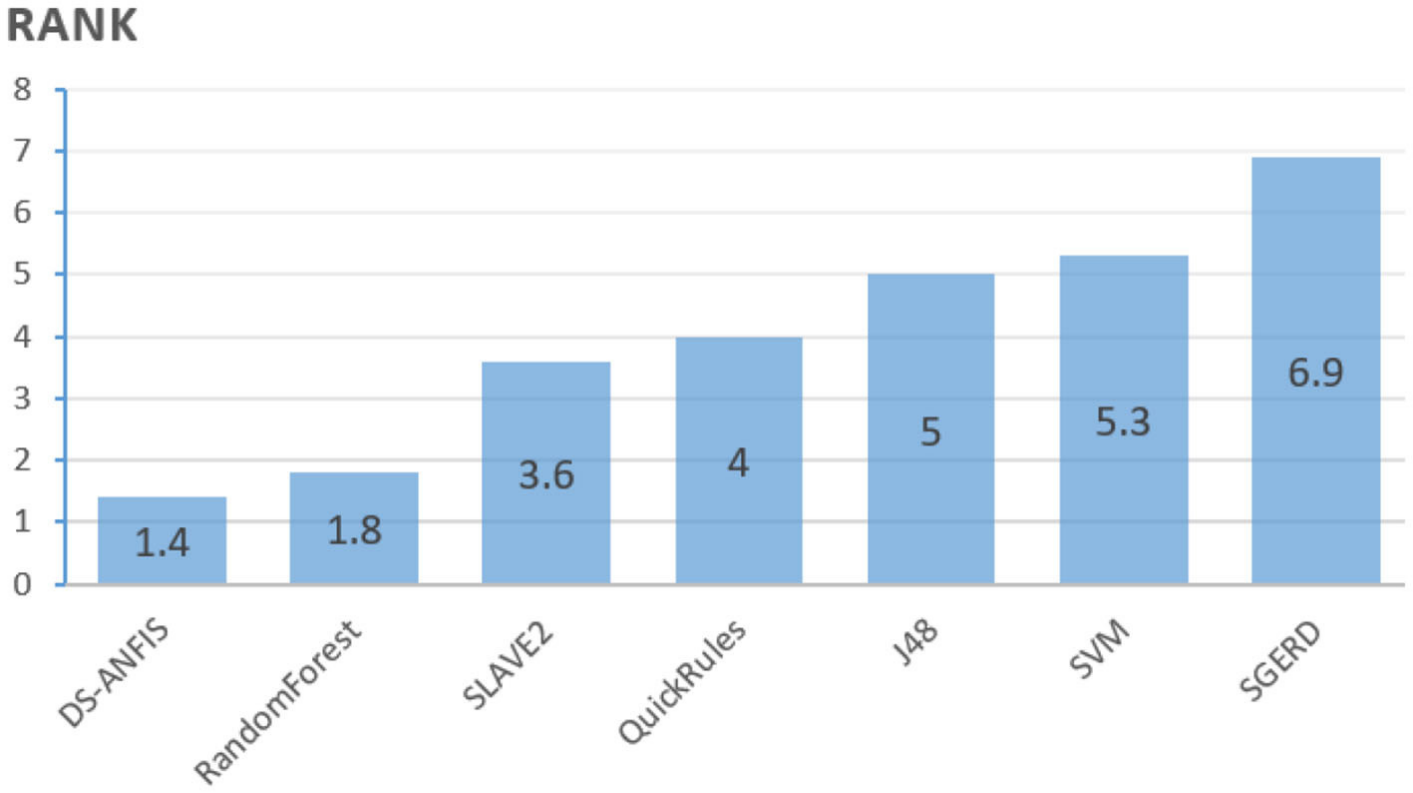
Ranks of algorithms.

Based on the results from Table 4, the Friedman test is employed resulting in the rankings as calculated in Figure 11, where the average ranking obtained for each algorithm are proportional to the bars. The lowest bar, which corresponds to the most powerful algorithm statistically, is consistent with the best averaged accuracies achieved by DS-ANFIS in Table 4. Experimentation continues to examine whether a statistically significant difference exists among the collection of errors through the Friedman test. The *p*-value, as shown in Table 5, is the probability that rejects the null hypothesis, i.e., no statistically significant difference occurs among the performances of the seven models. The null hypothesis can therefore be clearly rejected in Table 5, in the case of the significance level being α = 0.01, confirming the existence of such significant statistical differences in results obtained by these classifiers as a whole.

**Table 5:** Friedman test result.

**Comparison Pool**	**Hypothesis (α = 0.01)**	***p*-value**	**Statistic**
DS-ANFIS, J48, SVM, RandomForest, SGERD, SLAVE2, QuickRules	Reject	0.00000	40.02724

Despite the Friedman test enabling to detection of significant differences with respect to a collection of models as a whole, it does not support the explicit identification of comparisons when having a particular model as a control method against the remaining alternatives. As the proposed approach and the best performing classifier, it is of natural appeal to use DS-ANFIS as the control method in comparison to six competitors. The standard Finner's procedure (Demšar, 2006) is applied to run the test, calculating the adjusted *p*-values, with further results presented in Table 6. According to the *p*-values, the null hypothesis that there exists no significant performance difference between the proposed DS-ANFIS and SGERD, SVM, J48, QuickRules, or SLAVE2 is rejected at the level of significance specified by α = 0.05. In comparison with the powerful random forest, the conclusion of no statistical difference remains consistent with that of the previous *t*-test result. Despite the competitive performance of the random forest classifier, which is generally considered a black-boxed approach without interpretability (Song et al., 2013), the proposed method as demonstrated above is superior in providing explainability, which allows to track back how the dementia diagnosis may be achieved, markedly facilitating clinical decision support.

**Table 6:** Results of Finner's procedure with DS-ANFIS as the control method.

**Comparison**	**Statistic**	**Adjusted**	**Hypothesis**
		***p*-value**	**(α = 0.05)**
DS-ANFIS vs. SGERD	5.69304	0.00000	Rejected
DS-ANFIS vs. SVM	4.03688	0.00016	Rejected
DS-ANFIS vs. J48	3.72635	0.00039	Rejected
DS-ANFIS vs. QuickRules	2.69126	0.01066	Rejected
DS-ANFIS vs. SLAVE2	2.27722	0.02727	Rejected
DS-ANFIS vs. RandomForest	0.41404	0.67885	Accepted

To give an overview of the run time overheads executing the proposed method in practice, Table 7 summarizes the run time (in seconds) in comparison with the alternatives. The implementation of the proposed method was undertaken through Matlab run in a laptop configured with Intel i7-7500 CPU and 8192M RAM. It is worth noting that the proposed implementation was not purposefully optimized to achieve the best run time efficacy, therefore, the run time efficiency could potentially be further improved where a more carefully calibrated implementation is employed. In comparison with the fuzzy alternative SGERD with a cost of 2.12 s, DS-ANFIS executes the program with slightly more time of 2.79 s but with a significant performance gain of 81.69% over SGERD of 74.744% as previously summarized. The proposed algorithm is significantly faster compared to the popular SLAVE2 that is also implemented in KEEL software (Alcalá et al., 2010) like SGERD, which is possibly due to the SLAVE2 learns fuzzy rules employing the evolutionary algorithm that is population-based and takes longer to converge. The running of J48, SVM, RandomForest, and QuickRules generally takes less than 1 s, which is likely attributed to the use of the highly optimized WEKA (Witten et al., 2016) platform. Despite the differences in run time costs may partly result from the use of different implementation platforms and/or various level of code optimizations by different researchers, it's reasonable to conclude the run time overhead of the proposed method is efficient in practice that only costs less 0.3 s on average for a single execution.

**Table 7:** Run time (in seconds) comparison.

**Run**	**DS-ANFIS**	**J48**	**SVM**	**RandomForest**	**SGERD**	**SLAVE2**	**QuickRules**
1	3.34	0.12	0.17	0.25	1.72	32.12	1.00
2	2.79	0.08	0.09	0.17	1.94	55.20	0.88
3	2.67	0.06	0.07	0.12	2.08	43.60	0.94
4	2.67	0.02	0.08	0.14	1.90	44.99	0.78
5	2.69	0.04	0.07	0.18	1.88	45.94	0.87
6	2.78	0.04	0.12	0.13	2.25	41.98	0.76
7	2.59	0.00	0.06	0.12	2.24	34.50	0.72
8	2.84	0.04	0.06	0.18	2.78	36.01	0.86
9	2.68	0.02	0.06	0.14	2.29	33.20	0.80
10	2.85	0.04	0.09	0.14	2.18	34.91	0.84
Average	2.79 ± 0.21	0.05 ± 0.03	0.09 ± 0.03	0.16 ± 0.04	2.12 ± 0.30	40.25 ± 7.36	0.85 ± 0.08

## 5. Conclusion

This article has proposed a novel approach that learns an interpretable fuzzy rule-based system for clinical decision support of dementia diagnosis. It adapts the concept of dominant sets that arises from the study of graph theory and formulates the dementia diagnosis as a pairwise clustering problem. A peeling-off strategy is then used, which iteratively extracts from the constructed edge-weighted graph a dominant set, enabling it to automatically detect a fixed set of clusters. Each dominant set is then converted into a parameterized fuzzy rule, which is further optimized in a supervised adaptive network-based fuzzy inference framework.

The experimental results demonstrate an interpretable system comprised of human-readable rules, and how it may be employed to explain an overall decision by decomposing the contributions made by individual rules through a robust inference framework, thus facilitating clinical decision support. The interpretation is also supported by the generation of a reasonable rule base size consisting of fewer than 14 rules on average per fuzzy system. This is further supported by an illustrative representation of the learned knowledge and the reasoning process through the example in Section 4.1, which enhances the communication and delivery of these results to non-expert users. Further comparative studies have shown that the proposed work achieves statistically better or at least, comparable performance to state-of-the-art fuzzy and non-fuzzy alternatives.

Interesting future study remains for further development, despite promising results. This includes evaluating it on dementia data sets on a larger scale, especially where subjects are available across different severity levels for multi-class diagnosis and fine-tuning the ANFIS framework for further optimization. Finally, future studies will also include developing an integrated approach that enables the fuzzy system to directly work with missing values that commonly exist in dementia research.

## Data Availability Statement

Publicly available datasets were analyzed in this study. This data can be found at: https://www.oasis-brains.org/.

## Author Contributions

TC: conceptualization, formal analysis, literature research, methodology, validation, writing–draft, and review. PS: formal analysis, methodology, and writing–review. YS: formal analysis and writing–review and editing. LC, MM, YZ, and GA: writing–review and editing. All the authors contributed to the article and approved the submitted version.

## Funding

This study has been supported by grants from the National Natural Science Foundation of China (Nos. 61906181, 72001032, and 62003200), the China Postdoctoral Science Foundation (No. 2020M673148), and the Technological Innovation Programs of Higher Education Institutions in Shanxi (No. 2020L0016).

## Conflict of interest

The authors declare that the research was conducted in the absence of any commercial or financial relationships that could be construed as a potential conflict of interest. Reviewer BA declared a shared affiliation with the authors TC and GA at the time of review.

## Publisher's note

All claims expressed in this article are solely those of the authors and do not necessarily represent those of their affiliated organizations, or those of the publisher, the editors and the reviewers. Any product that may be evaluated in this article, or claim that may be made by its manufacturer, is not guaranteed or endorsed by the publisher.
